# The Feasibility of Examining the Effects of Gastric Bypass Surgery on Intestinal Metabolism: Prospective, Longitudinal Mechanistic Clinical Trial

**DOI:** 10.2196/12459

**Published:** 2019-01-24

**Authors:** Anita P Courcoulas, Margaret A Stefater, Eleanor Shirley, William F Gourash, Nicholas Stylopoulos

**Affiliations:** 1 University of Pittsburgh Medical Center Pittsburgh, PA United States; 2 Endocrinology Boston Children's Hospital Boston, MA United States

**Keywords:** bariatric surgery, obesity, diabetes mellitus, metabolism, gastric bypass, intestine, research subject recruitment, feasibility studies

## Abstract

**Background:**

Bariatric surgery, especially Roux-en-Y gastric bypass (RYGB), is the best treatment for severe obesity and its complications including type 2 diabetes mellitus (T2DM). Understanding the mechanisms responsible for the beneficial metabolic effects will help to engineer ways to improve the procedure or produce these effects without surgery.

**Objective:**

The aim is to present data on recruitment and feasibility of a translational study designed to collect intestinal samples before and after bariatric surgery. The goal of biobanking is to allow future studies to test the hypothesis that the mechanism of action of RYGB involves specific changes in the postsurgical short- and long-term metabolism and morphology of the jejunum (Roux limb). Specifically, to test whether the intestine enhances its metabolism and activity after RYGB and increases its fuel utilization, we designed a prospective, longitudinal study, which involved the recruitment of candidates for RYGB with and without T2DM. We describe the tissue bank that we have generated, and our experience, hoping to further facilitate the performance of longitudinal mechanistic studies in human patients undergoing bariatric surgery and especially those involving post-RYGB intestinal biology.

**Methods:**

We conducted a trial to characterize the effects of RYGB on intestinal metabolism. Intestinal tissue samples were collected from the jejunum at surgery, 1, 6, and 12 months postoperatively for the analysis of intestinal gene expression and metabolomic and morphologic changes. The target number of patients who completed at least the 6-month follow-up was 26, and we included a 20% attrition rate, increasing the total number to 32.

**Results:**

To enroll 26 patients, we had to approach 79 potential participants. A total of 37 agreed to participate and started the study; 33, 30, and 26 active participants completed their 1-month, 6-month, and 12-month studies, respectively. Three participants withdrew, and 30 participants are still active. Altruism and interest in research were the most common reasons for participation. Important factors for feasibility and successful retention included (1) large volume case flow, (2) inclusion and exclusion criteria broad enough to capture a large segment of the patient population but narrow enough to ensure the completion of study aims and protection of safety concerns, (3) accurate assessment of willingness and motivation to participate in a study, (4) seamless integration of the recruitment process into normal clinical flow, (5) financial reimbursement and nonfinancial rewards and gestures of appreciation, and (6) nonburdensome follow-up visits and measures and reasonable time allotted.

**Conclusions:**

Human translational studies of the intestinal mechanisms of metabolic and weight changes after bariatric surgery are important and feasible. A tissue bank with unique samples has been established that could be used by investigators in many research fields, further enabling mechanistic studies on the effects of bariatric surgery.

**Trial Registration:**

ClinicalTrials.gov NCT02710370; https://clinicaltrials.gov/ct2/show/NCT02710370 (Archived by WebCite at http://www.webcitation.org/75HrQT8Dl)

## Introduction

### Background

Several recent studies have concluded that bariatric surgery, especially Roux-en-Y gastric bypass (RYGB), is the best current treatment option for obesity and obesity-related comorbid conditions, including type 2 diabetes mellitus (T2DM) [1-3]. Although controversial, many investigators have advocated, based on many clinical observations as well as on findings of preclinical studies in animal models, that the effectiveness of RYGB does not depend upon body weight loss. Unraveling the mechanisms underlying the metabolic effects of weight loss surgery will help to engineer ways to improve the surgical procedures or to produce these effects without surgery. To this end, human translational studies will be required; however, a challenge hindering progress is the lack of knowledge about the feasibility of and strategy for recruiting participants for mechanistic studies that require potentially invasive methods.

### Objectives

The focus of this study is to present data on recruitment and feasibility of a translational study designed to collect intestinal samples before and after bariatric surgery. The goal of biobanking is to allow future studies to test the hypothesis that the mechanism of action of RYGB involves specific changes in the postsurgical short- and long-term metabolism and morphology of the jejunum (Roux limb) [4]. Specifically, the intestine enhances its metabolism and activity after gastric bypass, resulting in an increase in fuel utilization. This is manifested as augmented intestinal utilization of glucose, cholesterol, and amino acids, which might in turn improve whole-body metabolism and T2DM. To investigate this hypothesis in humans, studies had to be designed to recruit bariatric bypass surgery candidates with and without T2DM to participate in a longitudinal study protocol, which involved collection of intestinal tissue at the time of surgery and at later time points during the first year following surgery. We describe the tissue bank that we have generated, and we discuss in detail our experience, hoping to further facilitate the performance of longitudinal mechanistic studies in human patients undergoing bariatric surgery and especially those involving methods examining the postbypass intestinal biology.

## Methods

### Screening Strategy, Data Collection, and Outcome Measures

The inclusion and exclusion criteria of the study are shown in Textboxes 1 and 2 and were intentionally broad with respect to age and body mass index.

**Study inclusion criteria.**

Inclusion criteria

- Age ≥18 years who are to undergo Roux-en-Y gastric bypass
- Moderate to severe obesity: 35 > body mass index (BMI) ≤ 40 kg/m^2^ (with an obesity-related comorbidity) or BMI ≥ 40 kg/m^2^
- In total, 2 groups based on type 2 diabetes mellitus (T2DM) status:
  - No T2DM
  - T2DM confirmed by either a documented fasting blood glucose greater than 126 mg/dL, or hemoglobin A1c greater than or equal to 6.5, or treatment with an antidiabetic medication

**Study exclusion criteria.**

Exclusion criteria

- Prior bariatric or foregut surgery
- Unlikely to comply with follow-up protocol (eg, travel time from home to clinic too long to make visits feasible, unwilling to return for follow-up visits)
- Unable to communicate with local study staff (eg, foreign-language speaking persons who are unable to read, speak, or understand English well enough to participate)
- Known type 1 diabetes mellitus per the medical history
- Impaired mental status
- Drug and/or alcohol addiction
- Current smoking
- Portal hypertension and/or cirrhosis
- Coagulopathy
- Currently pregnant or plan to become pregnant in the next year

**Figure 1:**
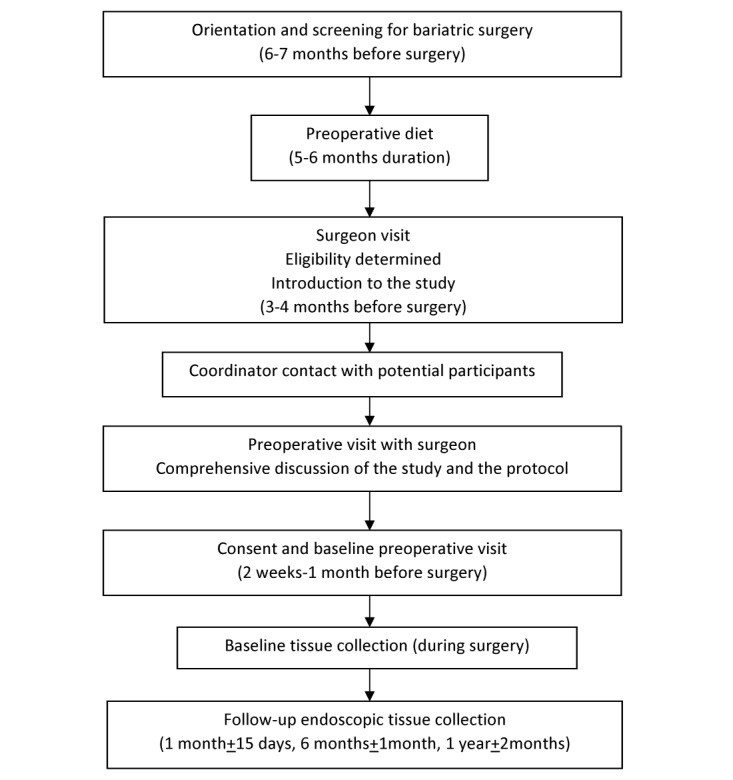
Study timeline.

The complete study timeline is shown in Figure 1. Potential bariatric surgery candidates per standard of clinical care attended an orientation session and completed a screening information form for bariatric surgery. They engaged in an insurance-required 5- to 6-month diet either through the bariatric group or with their family physician, nutritional evaluation by a dietitian, psychological evaluation that includes screening for substance abuse, and preoperative medical evaluation. Those with T2DM were referred for cardiac evaluation by a cardiologist. At 2 to 3 months into the diet, a one-on-one clinical visit with the surgeon and principal investigator (PI) was scheduled. The PI introduced and explained the research study and protocol to prospective patients if they met the inclusion criteria and did not meet the exclusion criteria (Textboxes 1 and 2). Written materials about the study were provided, and the study coordinator then contacted potential participants by phone to further discuss the study. After completing the required diet and obtaining insurance approval for their surgery, a preoperative visit was scheduled with the PI where a comprehensive review of the study protocol and participation was discussed. If the patient desired to be enrolled in the study, the consent process was completed and a baseline research visit was scheduled before the scheduled bariatric surgery. Baseline assessments were conducted within 30 days before the scheduled surgery, and baseline intestinal tissue samples were collected at the time of surgery. Follow-up assessments were conducted, and tissue samples were collected at 1, 6, and 12 months after bariatric surgery. The tissue samples were obtained via endoscopic biopsy performed by the surgeon of record from the RYGB. Additional baseline and follow-up assessments included laboratory tests (complete metabolic panel, complete blood count, hemoglobin A1c [HbA1c], and prothrombin time/international normalized ratio), physical measures (weight, percent body fat, neck, waist and hip circumference, blood pressure, and pulse), interviewer-administered forms (comorbidities, medication, and the Sigstad clinical diagnostic index), and self-report forms (demographic, eating and weight history, diabetes history, 36-item Short Form Health Survey [SF-36], additional treatments, glycemic symptoms, and the gastrointestinal and neurologic symptom forms). Comorbid conditions in addition to T2DM were determined using a combination of laboratory values, physical measures, patient reported medication use, and medical records review using standard definitions. As metformin is known to increase intestinal glucose utilization, patients on this medication were instructed not to take it 24 hours before their surgery and endoscopy. In addition, they were specifically asked about the exact time the last dose was taken before the operation or the endoscopy, and their response was documented. Health-related quality of life was measured using the Medical Outcomes Study SF-36.

The cost of all research-related activity including the endoscopies and biopsies and anesthesia care was funded by a research grant from the National Institute of Diabetes and Digestive and Kidney Diseases (NIDDK): grant R01DK108642. Emergency care for procedural complications would be paid by the study.

Study visits were completed in a Clinical and Translational Research Center located close to the outpatient clinic with on-site phlebotomy. Participants were provided parking (a voucher worth US $8) and remuneration for the baseline research visit (US $150) and an ascending remuneration for the 3 subsequent research visits, each of which included an endoscopy and biopsy (US $450, US $500, and US $600). Participants were also provided their anthropometric data and laboratory results at each visit, which graphically displayed their progress. Phone calls were made between in-person visits to foster study relationship and retention.

### Sample Size, Power, and Detectable Effects

Sample size calculations were performed using STATA/SE 15.0 (StataCorp LLC, College Station, TX) and G*Power (Heinrich-Heine-Universität Düsseldorf, Dusseldorf Germany) software, and it was determined that 26 individuals would need to be recruited within a 5-year period and complete at least the 1-month and the 6-month tissue collection. For the primary analyses, the main outcome measure will be the paired difference in gene expression levels in the same subject (before and each time point after RYGB; two-tailed, paired samples *t* tests). This is a design that maximizes power and minimizes variability. Overall, 1 published study that examined proliferation (measured as Ki-67 positive cells) in intestinal samples collected at the time of surgery and 8 months after RYGB [5] showed that the effect of RYGB on proliferation was so profound that statistically significant differences could be detected with a sample size of 8 participants. Our study involved more and different outcomes. We ran several scenarios for many genes based on the following input data: (1) significance level of .05, (2) power at least 80%, and (3) estimates of the expected mean and SD of the differences in gene expression levels. The estimates of the expected mean difference and the SD of the differences in expression levels were based on an initial pilot microarray study, which determined the gene expression levels in available samples from patients with RYGB and controls. For the sample size calculations, we first corrected for multiple hypothesis testing. The significance level cutoff was determined after controlling the false discovery rate (FDR). Specifically, if we choose a level of FDR *α=* 5% and we suppose that the proportion of the genes that will not be differentially expressed is *π*_*0*_*=* 90%, the adjusted *P* value cutoff *Λ* could be estimated by the formula: *Λ=* (*a*/1−*a*) * (1−π_0_/π_0_) [6]. On the basis of these assumptions, the adjusted cutoff would be .0058.

**Figure 2:**
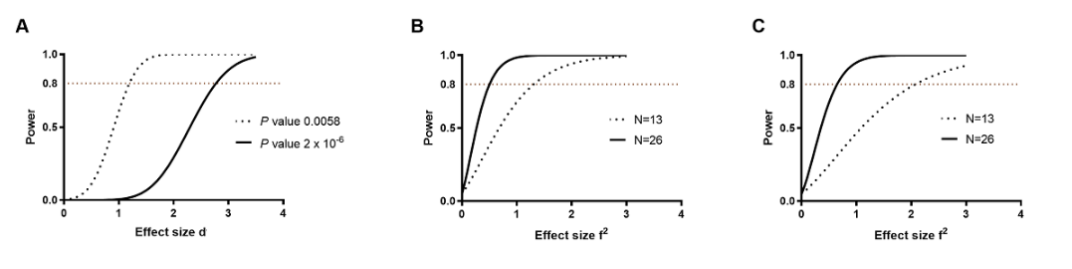
Power analysis. A. Curves of effect size (Cohen d), against power for paired samples t test. The effect size is the ratio between the mean of a difference divided by the SD of the difference in a study variable between the 2 groups (eg, here we used the Log2(fold change) in the expression levels of a gene). The power analysis was based on two adjusted P values: *P*=.0058 when controlling for FDR at 5% and *P*=2x10−6 when controlling for familywise error rate at 5%. B and C. Power analysis for regression modeling with 3 predictors (B) and 5 predictors (C). The effect size f2 is given by the ratio R2/(1−R2). R2 is the coefficient of determination. The following conventional values for the effect size f2 have been proposed: small f2=0.02, medium f2=0.15, large f2=0.35. We calculated the power for 2 sample sizes to determine the power of models that are based only on patients with or without diabetes (N=13) or the entire group (N=26). In all panels, the generally accepted cutoff of 80% for the power is shown.

We then can construct the curves of Cohen effect size *d* (mean/SD) against power (Figure 2). To this end, we use a cutoff of 33% as a meaningful change (increase or decrease) of the expression levels of a gene. On the basis of our pilot dataset, for all these genes that changed over 33%, the mean difference (in Log_2_[Fold change]) was 61%, and the SD of the differences was 41%. To determine the sample size to achieve 80% power, the following values were considered: significance level 0.0058, power 0.8, mean of differences for null hypothesis 0, mean of differences for alternative hypothesis 0.61 and SD of differences 0.41. On the basis of these estimates, we concluded that these analyses can generate meaningful results with 13 subjects. As we would like to study the effects of RYGB in patients with and without T2DM, we would need to double this estimate and recruit approximately 26 patients for this study. We also included a 20% attrition rate and thus the maximum number of patients for this study was increased to 32. The second and more stringent approach we explored was to calculate the cutoff for the type I error (ie, the *P* value) that should be accepted if we wanted to keep the familywise error rate (FWER) lower than 5%. For n=24,000 different comparisons (number of probes tested), the adjusted *P* value *β* can be calculated by the equation: *β*=1−(1− *FWER)*^*1/n*^ [7]. Thus, for our chosen FWER cutoff, differences in gene expression levels with a *P* value lower than 2 × 10^−^^6^ could be considered statistically significant. The curves of Cohen effect size *d* (mean/SD) against power while controlling for this value are shown in Figure 2.

Repeated measures analysis of variance could be used to evaluate changes among the 3 postoperative time points. To determine the power, we could achieve by including a single group of 13 patients in the analysis, we used GLIMMPSE software [8]. Assumptions used a significance level of .05 and the base case scenario was a parameter (gene expression levels) change of 33% at the 1-month time point and 50% at the 12-month time point post-RYGB. We varied the correlation between the measurements, and many scenarios were evaluated with the basic assumption being that the correlation is higher between the 1-month time point and 6-month time point than the 1-month time point and 12-month time point after RYGBS; the correlation is even higher between the 6-month time point and 12-month time point after RYGBS. We used the Hotelling-Lawley Trace test, which showed that power would be over 0.8 for the base assumptions. We also found that this sample size would provide adequate power to allow us to determine whether the 6-month time point and 12-month time point measurements are different from the 1-month time point measurements and whether the changes follow polynomial trends. For the power analysis of multiple linear regression models, we used 2 sample sizes (N=13 and N=26), and the power calculations are summarized in Figure 2.

## Results

### Recruitment and Retention Results

**Figure 3:**
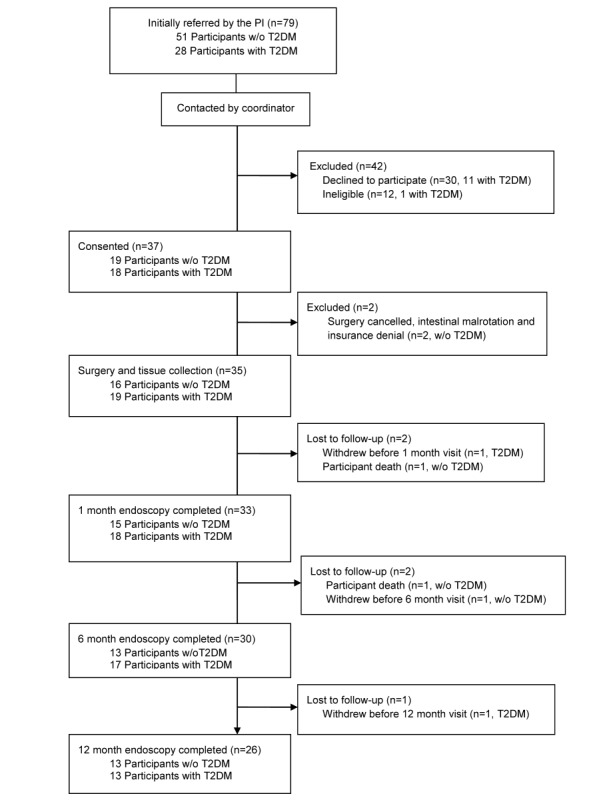
Consort diagram. 1 participant was reassigned to group T2DM based on her baseline blood work. PI: principal investigator; T2DM: type 2 diabetes mellitus; w/o: without.

The 26 participants who were required for this study were recruited and completed the assessments within 2 years and 8 months (from February 2016 to October 2018). In this period, 79 patients were determined to meet the criteria and were invited to participate in the study. Of the 79 potential participants, 30 declined to participate and 12 were found to be ineligible. Of these, 1 was subsequently found to be ineligible because of current smoking, 1 participant’s lab results did not document diabetes, and 10 did not complete the presurgical process before the recruitment had ended. Of the 30 candidates who declined participation, 11 decided not to undergo bariatric surgery at all, 3 chose to undergo sleeve gastrectomy rather than gastric bypass, 7 were concerned about missing additional work days, 3 felt travel time to the hospital would be burdensome, 2 were concerned about arranging for child care during their study visits, 2 were concerned about anesthesia and undergoing the endoscopic procedures, and 2 did not indicate a specific reason (Figure 3).

**Figure 4:**
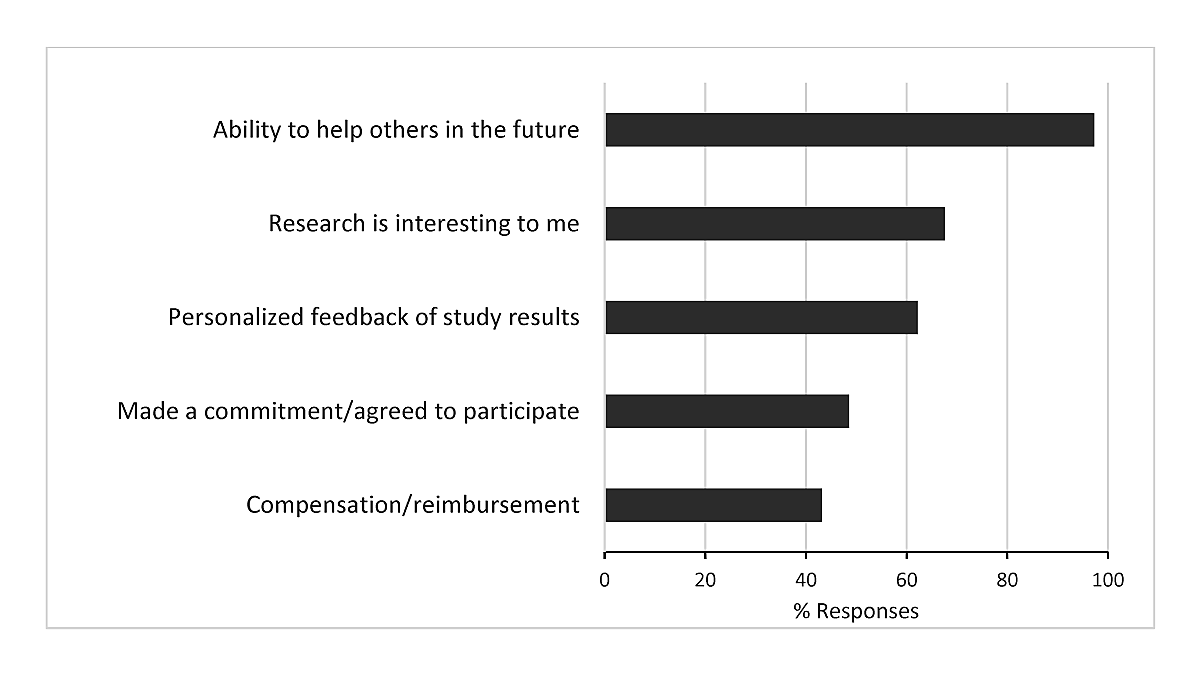
Participants’ reasons for study enrollment. N=37: 36 endorsed helping others, 25 reported that research is interesting, 23 reported that personalized feedback was helpful, 18 reported that they had made a commitment, and 16 reported that compensation influenced their decision to enroll in the study.

After the consent, 1 participant’s surgical procedure was aborted because of intestinal malrotation, and the participant was inactivated. Another participant’s surgery was cancelled, as her insurance did not authorize her procedure. There were 3 withdrawals during the study follow-up. One of the participants with T2DM chose to withdraw after her surgery, before her 1-month assessment. A participant without T2DM chose to withdraw before her 6-month visit, and a participant with T2DM chose to withdraw before his 12-month visit. There were 2 participant deaths (both without T2DM, 1 before the 1-month follow-up from a cardiac event, and 1 before 6-months from possible substance use). When the consented participants were asked on the 9-item self-report survey to choose the reasons that contributed to their participating in the study, 36 participants (97%, 36/37) chose the “ability to help others in the future,” 25 participants (67%, 25/37) chose that “research is interesting to me,” 23 (62%, 23/37) chose the “personalized feedback of study results,” 18 (48%, 18/37) chose they “made a commitment/agreed to participate,” and 16 (43%, 16/37) chose the “compensation/reimbursement.” (Figure 4) A list of the comorbidities and medications used by the participants at the time of their surgery are listed in Tables 1 and 2.

Recruitment ended after 26 patients completed the 12-month follow-up. At that time, a total of 35 patients had been recruited and had their initial tissue collection during the RYGB surgery. In terms of study retention, 33 active participants have completed their 1-month visit and endoscopic tissue collection (15 controls and 18 participants with T2DM) or 100% of the cohort (Figure 3); 30 participants completed a 6-month visit and 26 (the study target) of the 12-month visits were completed. The attrition at 12 months was 5 of 31 visits or 16.1 % (2 participants who did not have a surgical procedure and 4 participants who have not yet completed their 12-month visit were excluded from this total). The typical clinical attrition in our bariatric surgical program at 12 months for RYGB patients is higher at 25%.

A first analysis using samples from this study has been published recently [9]. The tissue samples or biopsies that were collected will be used for intestinal gene and protein expression, metabolite profile, and assessment of morphologic changes. Plasma and serum samples are also analyzed in parallel. Blood metabolomic signatures can contextualize intestinal tissue metabolomic data to enhance the understanding of intestinal energy utilization after RYGB. These signatures can also be correlated with clinical outcomes such as HbA1c, fasting glucose levels, or body weight loss.

The power and sample size calculations have been presented above in detail. Adjustment for multiple comparisons will be based on the FDR procedure by Benjamini and Yekutieli, allowing for between-metabolite correlations [10]. We will verify the Benjamini and Yekutieli FDR by comparing it with the empirical FDR using the permutation-based approach [11].

For the analysis of the relationship between clinical outcomes and the gene expression or metabolomic signatures, the number of covariates that can be included in the models will need to be limited to preserve degrees of freedom and avoid overfitting (Figure 2). One approach will be to decrease the dimensionality of the data by performing a principal component analysis, as correlation between gene or metabolite levels is expected, given that many of these reside in overlapping pathways. Another analytical approach will be to use Bayesian statistical methods that do not depend on sample size.

**Table 1:** Patient comorbidities (N=26).

Comorbidity		Patients, n
**Hypertension**		
	Borderline no medications	5
	Treatment with 1 medication	2
	Treatment with multiple medication	6
**Peripheral vascular disease**		
	Stroke, loss of tissue because of ischemia	1
**Lower extremity edema**		
	Intermittent lower extremity edema, not requiring treatment	3
	Symptoms requiring treatment, diuretics, elevation, or hose	3
**Deep vein thrombosis (DVT)/Pulmonary embolism (PE)**		
	History of DVT resolved with anticoagulation	1
	Previous PE	1
**Glucose metabolism**		
	Elevated fasting glucose	2
	Diabetes, controlled with oral medication	4
	Diabetes, controlled with insulin and oral medication	4
	Diabetes, with severe complications (retinopathy, neuropathy, renal failure, and blindness)	3
**Lipids (dyslipidemia or hyperlipidemia)**		
	Present, no treatment required	3
	Controlled with single medication	12
**Obstructive sleep apnea syndrome**		
	Sleep apnea symptoms (negative sleep study or not done)	3
	Sleep apnea diagnosis by sleep study (no oral appliance)	2
	Sleep apnea requiring oral appliance such as continuous positive airway pressure machine	14
**Asthma**		
	Intermittent mild symptoms, no medication	2
	Symptoms controlled with oral inhaler	3
**Gastroesophageal reflux disease**		
	Intermittent or variable symptoms, no medication	3
	Intermittent medication	1
	Histamine H2-receptor antagonists-H2 blockers or low-dose proton pump inhibitors (PPIs)	6
	High-dose PPI	3
**Cholelithiasis**		
	Gallstones with no symptoms	1
	Gallstones with severe symptoms or history of cholecystectomy	10
**Liver disease**		
	Modest or greater hepatomegaly liver function test alteration, fatty change category 2	4
**Back pain**		
	Intermittent symptoms not requiring medical treatment	6
	Symptoms requiring non-narcotic treatment	2
	Degenerative changes or positive objective findings, symptoms requiring narcotic treatment	2
**Musculoskeletal disease**		
	Pain with community ambulation	2
	Non-narcotic analgesia required	6
	Pain with household ambulation	1
	Awaiting or past joint replacement or other disability	1
**Fibromyalgia**		
	Treatment with non-narcotic medications	4
**Polycystic ovary syndrome (PCOS)**		
	Symptoms of PCOS, no treatment	5
	Oral contraceptive pills or antiandrogen prescription (Rx)	1
	Metformin or Thiazolidinediones	1
**Menstrual irregularities**		
	Irregular periods or oligomenorrhea	6
	Prior total abdominal hysterectomy	3
**Mental health diagnosis**		
	Bipolar disorder	1
	Anxiety and panic disorder	12
**Psychosocial impairment**		
	Mild impairment in psychosocial functioning but able to perform all primary tasks	5
	Moderate impairment in psychosocial functioning but able to perform most primary tasks	1
	Moderate impairment in psychosocial functioning and unable to perform some primary tasks	1
**Depression**		
	Mild and episodic not requiring treatment	1
	Moderate, accompanied by some impairment, may require treatment	6
	Moderate, with significant impairment, treatment indicated	9
**Stress Urinary Incontinence**		
	Minimal and intermittent	3
**Abdominal hernia**		
	Asymptomatic hernia, no prior operation	1
**Abdominal skin pannus**		
	Intertriginous irritation	1
**Smoking status**		
	Current smoker	1
	Former smoker (average 15.3 pack-years)	10

**Table 2:** List of medications used by the study participants at the time of their surgery (N=26).

Medication		Patients, n
**Antidiabetic**		
	Metformin	11
	Insulin	6
	Glipizide	2
	Repaglinide	2
	Liraglutide	2
	Dulaglutide	1
	Linagliptin	1
	Sitagliptin	1
	Empagliflozine	1
	Insulin glargine	1
**Nonsteroidal anti-inflammatory drugs**		
	Acetylsalicylic acid	5
	Ibuprofen	3
	Meloxicam	2
	Indomethacin	1
	Diphenhydramine and acetaminophen	1
**Antihypertensive**		
	Amlodipine	5
	Hydrochlorothiazide	4
	Lisinopril	3
	Atenolol	2
	Diltiazem	2
	Ramipril	1
	Losartan	1
	Irbesartan	1
	Valsartan	1
	Labetalol	1
	Prazosin	1
	Furosemide	1
	Hydrochlorothiazide/triamterene	1
**Oral birth control**		
	Norgestimate	3
	Levonorgestrel	1
	Medroxyprogesterone	1
**Statins and fibrates**		
	Atorvastatin	5
	Simvastatin	2
	Rosuvastatin	2
	Gemfibrozil	2
	Fenofibrate	1
	Ezetimibe	1
**Proton pump inhibitors** **and** **Histamine H2-receptor antagonists-H2 antagonists**		
	Omeprazole	5
	Pantoprazole	3
	Ranitidine	2
	Lansoprazole	2
	Loratadine	1
**Antidepressants and central nervous system-acting**		
	Gabapentin	6
	Sertaline	4
	Clonazepam	3
	Citalopram	2
	Topiramate	2
	Amytriptiline	2
	Paroxetine	1
	Escitalopram	1
	Fluoxetine	1
	Desvenlafaxine	1
	Quetiapine	1
	Trazodone	1
	Olanzepine	1
	Ziprasidone	1
	Risperidone	1
	Prochlorperazine	1
	Lorazepam	1
	Buproprion	1
	Buspirone	1
	Zolpidem	1
	Melatonin	1
	Venlafaxine	1
	Duloxetine	2
	Mirtazapine	1
	Oxycodone	1
**Antiallergic**		
	Fluticasone	3
	Hydroxyzine	1
	Diphenydramine	1
	Promethazine	1
**Other**		
	Levothyroxine	7
	Albuterol	3
	Cyclobenzaprine	3
	Pramipexole	1
	Iodine	1
	Linaclotide	1
	Sulfasalazine	1
	Infliximab	1
	Cyclosporine	1
	Vardenafil	1
	Rivaroxaban	1
	Docusate	1
	Erythromycin	1
	Botox	1
	Valacyclovir	1
	Nystatin	1
**Vitamins and supplements**		
	Multivitamin	6
	B and B12	6
	Omega-3 fatty acids	2
	D3	2
	Folic acid	1
	Biotin	1
	C	1
	Ferrous	1
	Calcium	1
	Potassium	1
	Chromium	1
	Turmeric curcumin	1
	Glucosamine chondroitin	1
	Fish oil	1
	Calcium carbonate	1
	Magnesium oxide	1
	Probiotic	1

### Lessons Learned and Important Factors for Feasibility and Successful Retention

We approached patient recruitment in a systematic way using a published conceptual framework for study feasibility, which comprises determining, operationalizing, and adequately resolving a series of essential factors. [12] The first factor was the number of potentially eligible participants who needed to be considered. For bariatric surgery, the presurgical work-up and insurance approval steps are both rigorous and time-consuming, so the potential candidates will often discontinue the process. Many candidates had to be considered and approached at a time in the process where their certainty of ultimately undergoing surgery was relatively high. Typically, for bariatric surgery patients, this time frame was around 3 months into a commonly required 5- to 6-month presurgical preparation period. Therefore, it was essential to target a practice large enough to ensure a large volume or flow of patients who met the eligibility criteria and could be approached for recruitment. The second factor was that the inclusion and exclusion criteria had to be broad enough to capture a large segment of the patient population to approach but narrow enough to ensure the completion of study aims and protection of safety concerns. The third factor was an accurate assessment of the willingness and motivation of people to participate in a study. We conducted an informal pilot project before engaging in the final study and surveyed gastric bypass candidates in person at their clinic evaluation about their potential willingness to participate in a study such as this one. We found that most candidates were agreeable to the idea, motivated by altruism, and interested in understanding the mechanism of diabetes improvement. In the actual study recruitment process, those with T2DM or a family history of T2DM were particularly motivated to contribute to understanding the underlying mechanisms to help potentially affect a less invasive treatment or cure. Therefore, explaining in lay terms, the scientific basis and hypotheses was an important contributing factor to successful recruitment. The fourth factor was the actual recruitment process whereby and how participants were engaged into the study. Our experience suggests this should be incorporated into the normal clinical flow and integrated into the candidate’s experience so that it becomes a seamless part of a whole evaluation and participation scheme for an individual. This is also consistent with recruitment in pragmatic clinical trials where the conduct of research is integrated into the delivery of health care. [13] The fifth factor was that participants were pleased with any financial reimbursement for their expenses that included travel, parking, missed work, child care, and others. Nonfinancial rewards such as gestures of appreciation, reports of their progress, and between-visit calls were also very helpful and meaningful to participants. Finally, for a longitudinal study to be successful, complete retention of participants over time was needed. For this study, this meant that the study and clinic visits needed to be conducted efficiently, and at times, both research and clinical visits were done during the same encounter. Attention was also paid to the protocol design so that the visits and measures were nonburdensome and the time allotted was reasonable (maximum of 3-4 hours).

There were 3 additional factors specific to bariatric surgery that also might have played a role in the successful recruitment and retention into this study. The first important factor was that the surgeon of record for the original gastric bypass surgery carried out both the follow-up visits and the endoscopic tissue biopsies. Participants were comforted by the idea that their surgeon, who knew their anatomy, would be performing biopsies and simultaneously checking for anatomical problems. Participants had voiced, when asked, that being referred to another provider to gather the tissue samples was much less ideal because of their unfamiliarity and discomfort, especially at the early time points following surgery. The second factor was that for bariatric surgery clinical practice, completeness of follow-up in the first 12 months after surgery is typically high (83%-100%) [14]. Therefore, a longitudinal study that is completed within the first 12 months postoperatively for follow-up has a higher chance of success, as study retention rates decline dramatically after the first year, as does clinical follow-up [14]. The third factor was that preoperative bariatric surgery candidates expect and are very compliant with a lengthy and complex work-up process for the surgery itself, so they perhaps tend to be more willing to undertake extra research visits and testing than other types of surgery patients.

## Discussion

### Unanswered Questions and Future Research

Though many bariatric surgery candidates exhibit a willingness to participate in research, this willingness decreases when the research includes either invasive activities or longitudinal follow-ups [15]. Despite these limitations, the Longitudinal Assessment of Bariatric Surgery study, a prospective observational study, recruited 5108 participants over a 2-year time period for 30-day safety outcomes, and 2458 participants over 3 years for a more extensive study protocol, which was conducted during annual, in-person visits and included measurements, phlebotomy, a corridor walk, physical activity monitoring, and an extensive set of questionnaires [16-18]. Randomized controlled trials in bariatric surgery, particularly those that compare surgical with nonsurgical treatments, also pose recruitment challenges because of several issues: the existence of genuine clinical equipoise between the alternatives, participants not agreeing to a nonsurgical arm after spending a lifetime in the “nonintervention arm,” payers not funding the surgical procedures under study, and ethical issues with informed and voluntary consent [19,20]. Prospective clinical trials have been performed that include invasive procedures such as tissue biopsies at the time of the initial bariatric surgery (Table 1). Some of these studies have also obtained longitudinal samples over time, but these were typically collected at convenient time points, when other surgical procedures were done for a clinical indication (eg, for management of surgical complications and cholecystectomy) and not at protocol-specified intervals. Moreover, most studies have utilized tissue sampled from adipose tissue, skeletal muscle, and liver, which is readily available via percutaneous biopsy (Multimedia Appendix 1 [5,21-59]). Prospective, longitudinal studies that seek to explore mechanistic goals by collecting and analyzing tissue from intra-abdominal tissues and organs are virtually absent in the literature.

A review of the Gene Expression Omnibus database revealed only a few datasets that include gene expression profiles of tissues after weight loss surgery [21,22,60-62]. In addition, there are only a few studies that use invasive means to interrogate the metabolic perturbations of weight loss surgery. An exception is the use of hyperinsulinemic-euglycemic clamping. However, although this method is considered the gold standard for estimation of insulin sensitivity, many studies disagree on the role of hepatic and peripheral insulin sensitivity in contributing to diabetes remission after surgery. Although some studies suggest an early role for improved hepatic [63] or peripheral [64,65] insulin sensitivity in contributing to glycemic improvement in the first weeks after surgery, others do not report this effect [66-69].

### Conclusions

As this exciting research field of bariatric surgery mechanisms continues to grow, the need for further invasive studies in humans will continue to grow as well. We demonstrate that translational longitudinal clinical trials on intestinal mechanisms are both important and feasible, and we share lessons learned regarding participant recruitment and retention. Factors for successful recruitment and retention included large volume case flow, broad inclusion criteria, integrating study and clinical procedures, participant reimbursement or remuneration, sharing test or measurement data, and minimizing study burden. We believe that the established biobank could further facilitate studies examining the effects of bariatric surgery on intestinal biology and we hope that the factors discussed in this study appear to inform and support successful recruitment and retention into these unique types of trials.

## Appendix

Multimedia Appendix 1 Bariatric surgery studies involving analyses of tissue biopsies.

## Abbreviations

BMI: body mass index
DVT: deep vein thrombosis
FDR: false discovery rate
FWER: familywise error rate
HbA1c: hemoglobin A1c
NIDDK: National Institute of Diabetes and Digestive and Kidney Diseases
NIH: National Institutes of Health
PCOS: polycystic ovary syndrome
PE: pulmonary embolism
PI: principal investigator
PPIs: proton pump inhibitors
RYGB: Roux-en-Y gastric bypass
SF-36: 36-item Short Form Health Survey
T2DM: type 2 diabetes mellitus

## References

[1] Rubino F, Nathan DM, Eckel RH, Schauer PR, Alberti KG, Zimmet PZ, Del Prato S, Ji L, Sadikot SM, Herman WH, Amiel SA, Kaplan LM, Taroncher-Oldenburg G, Cummings DE, Delegates of the 2nd Diabetes Surgery Summit (2016). Metabolic surgery in the treatment algorithm for type 2 diabetes: a joint statement by international diabetes organizations. Diabetes Care.

[2] Schauer PR, Bhatt DL, Kirwan JP, Wolski K, Aminian A, Brethauer SA, Navaneethan SD, Singh RP, Pothier CE, Nissen SE, Kashyap SR, STAMPEDE Investigators (2017). Bariatric surgery versus intensive medical therapy for diabetes - 5-year outcomes. N Engl J Med.

[3] Sjöström L, Peltonen M, Jacobson P, Ahlin S, Andersson-Assarsson J, Anveden A, Bouchard C, Carlsson B, Karason K, Lönroth H, Näslund I, Sjöström E, Taube M, Wedel H, Svensson PA, Sjöholm K, Carlsson LM (2014). Association of bariatric surgery with long-term remission of type 2 diabetes and with microvascular and macrovascular complications. J Am Med Assoc.

[4] Saeidi N, Meoli L, Nestoridi E, Gupta NK, Kvas S, Kucharczyk J, Bonab AA, Fischman AJ, Yarmush ML, Stylopoulos N (2013). Reprogramming of intestinal glucose metabolism and glycemic control in rats after gastric bypass. Science.

[5] Spak E, Björklund P, Helander HF, Vieth M, Olbers T, Casselbrant A, Lönroth H, Fändriks L (2010). Changes in the mucosa of the Roux-limb after gastric bypass surgery. Histopathology.

[6] Liu P, Hwang JT (2007). Quick calculation for sample size while controlling false discovery rate with application to microarray analysis. Bioinformatics.

[7] Lee ML, Whitmore GA (2002). Power and sample size for DNA microarray studies. Stat Med.

[8] Kreidler Sm, Muller KE, Grunwald GK, Ringham BM, Coker-Dukowitz ZT, Sakhadeo UR, Barón AE, Glueck DH (2013). GLIMMPSE: online power computation for linear models with and without a baseline covariate. J Stat Softw.

[9] Ben-Zvi D, Meoli L, Abidi WM, Nestoridi E, Panciotti C, Castillo E, Pizarro P, Shirley E, Gourash WF, Thompson CC, Munoz R, Clish CB, Anafi RC, Courcoulas AP, Stylopoulos N (2018). Time-dependent molecular responses differ between gastric bypass and dieting but are conserved across species. Cell Metab.

[10] Yekutieli D, Benjamini Y (2001). The control of the false discover rate in multiple testing under dependency. Ann Stat.

[11] Dudbridge F (2006). A note on permutation tests in multistage association scans. Am J hum genet.

[12] Eldridge SM, Lancaster GA, Campbell MJ, Thabane L, Hopewell S, Coleman CL, Bond CM (2016). Defining feasibility and pilot studies in preparation for randomised controlled trials: development of a conceptual framework. PLoS One.

[13] Oude Rengerink K, Kalkman S, Collier S, Ciaglia A, Worsley SD, Lightbourne A, Eckert L, Groenwold RH, Grobbee DE, Irving EA, Work Package 3 of the GetReal consortium (2017). Series: pragmatic trials and real world evidence: paper 3. Patient selection challenges and consequences. J Clin Epidemiol.

[14] Garb J, Welch G, Zagarins S, Kuhn J, Romanelli J (2009). Bariatric surgery for the treatment of morbid obesity: a meta-analysis of weight loss outcomes for laparoscopic adjustable gastric banding and laparoscopic gastric bypass. Obes Surg.

[15] Tichansky DS, Madan AK, Ternovits CA, Fain JN, Kitabchi AE (2007). Laparoscopic bariatric patients' will to help: the foundation of research. Surg Obes Relat Dis.

[16] Belle SH, Berk PD, Chapman WH, Christian NJ, Courcoulas AP, Dakin GF, Flum DR, Horlick M, King WC, McCloskey CA, Mitchell JE, Patterson EJ, Pender JR, Steffen KJ, Thirlby RC, Wolfe BM, Yanovski SZ, LABS Consortium (2013). Baseline characteristics of participants in the Longitudinal Assessment of Bariatric Surgery-2 (LABS-2) study. Surg Obes Relat Dis.

[17] Flum DR, Belle SH, King WC, Wahed AS, Berk P, Chapman W, Pories W, Courcoulas A, McCloskey C, Mitchell J, Patterson E, Pomp A, Staten MA, Yanovski SZ, Thirlby R, Wolfe B, Longitudinal Assessment of Bariatric Surgery (LABS) Consortium (2009). Perioperative safety in the longitudinal assessment of bariatric surgery. N Engl J Med.

[18] Courcoulas AP, Christian NJ, Belle SH, Berk PD, Flum DR, Garcia L, Horlick M, Kalarchian MA, King WC, Mitchell JE, Patterson EJ, Pender JR, Pomp A, Pories WJ, Thirlby RC, Yanovski SZ, Wolfe BM, Longitudinal Assessment of Bariatric Surgery (LABS) Consortium (2013). Weight change and health outcomes at 3 years after bariatric surgery among individuals with severe obesity. J Am Med Assoc.

[19] Courcoulas AP, Goodpaster BH, Eagleton JK, Belle SH, Kalarchian MA, Lang W, Toledo FG, Jakicic JM (2014). Surgical vs medical treatments for type 2 diabetes mellitus: a randomized clinical trial. JAMA Surg.

[20] Paramasivan S, Rogers CA, Welbourn R, Byrne JP, Salter N, Mahon D, Noble H, Kelly J, Mazza G, Whybrow P, Andrews RC, Wilson C, Blazeby JM, Donovan JL (2017). Enabling recruitment success in bariatric surgical trials: pilot phase of the By-Band-Sleeve study. Int J Obes (Lond).

[21] Ahrens M, Ammerpohl O, von Schönfels W, Kolarova J, Bens S, Itzel T, Teufel A, Herrmann A, Brosch M, Hinrichsen H, Erhart W, Egberts J, Sipos B, Schreiber S, Häsler R, Stickel F, Becker T, Krawczak M, Röcken C, Siebert R, Schafmayer C, Hampe J (2013). DNA methylation analysis in nonalcoholic fatty liver disease suggests distinct disease-specific and remodeling signatures after bariatric surgery. Cell Metab.

[22] Park JJ, Berggren JR, Hulver MW, Houmard JA, Hoffman EP (2006). GRB14, GPD1, and GDF8 as potential network collaborators in weight loss-induced improvements in insulin action in human skeletal muscle. Physiol Genomics.

[23] Ranløv I, Hardt F (1990). Regression of liver steatosis following gastroplasty or gastric bypass for morbid obesity. Digestion.

[24] Friedman JE, Dohm GL, Leggett-Frazier N, Elton CW, Tapscott EB, Pories WP, Caro JF (1992). Restoration of insulin responsiveness in skeletal muscle of morbidly obese patients after weight loss. Effect on muscle glucose transport and glucose transporter GLUT4. J Clin Invest.

[25] de Almeida SR, Rocha PR, Sanches MD, Leite VH, da Silva RA, Diniz MT, Diniz Mde F, Rocha AL (2006). Roux-en-Y gastric bypass improves the nonalcoholic steatohepatitis (NASH) of morbid obesity. Obes Surg.

[26] Klein S, Mittendorfer B, Eagon JC, Patterson B, Grant L, Feirt N, Seki E, Brenner D, Korenblat K, McCrea J (2006). Gastric bypass surgery improves metabolic and hepatic abnormalities associated with nonalcoholic fatty liver disease. Gastroenterology.

[27] Furuya Jr CK, de Oliveira CP, de Mello ES, Faintuch J, Raskovski A, Matsuda M, Vezozzo DC, Halpern A, Garrido Jr AB, Alves VA, Carrilho FJ (2007). Effects of bariatric surgery on nonalcoholic fatty liver disease: preliminary findings after 2 years. J Gastroenterol Hepatol.

[28] Gastaldi G, Russell A, Golay A, Giacobino JP, Habicht F, Barthassat V, Muzzin P, Bobbioni-Harsch E (2007). Upregulation of peroxisome proliferator-activated receptor gamma coactivator gene (PGC1A) during weight loss is related to insulin sensitivity but not to energy expenditure. Diabetologia.

[29] Sainsbury A, Goodlad RA, Perry SL, Pollard SG, Robins GG, Hull MA (2008). Increased colorectal epithelial cell proliferation and crypt fission associated with obesity and Roux-en-Y gastric bypass. Cancer Epidemiol Biomarkers Prev.

[30] Gregor MF, Yang L, Fabbrini E, Mohammed BS, Eagon JC, Hotamisligil GS, Klein S (2009). Endoplasmic reticulum stress is reduced in tissues of obese subjects after weight loss. Diabetes.

[31] Savu MK, Phillips SA, Oh DK, Park K, Gerlan C, Ciaraldi TP, Henry RR (2009). Response of adiponectin and its receptors to changes in metabolic state after gastric bypass surgery: dissociation between adipose tissue expression and circulating levels. Surg Obes Relat Dis.

[32] Kant P, Sainsbury A, Reed KR, Pollard SG, Scott N, Clarke AR, Coletta PL, Hull MA (2011). Rectal epithelial cell mitosis and expression of macrophage migration inhibitory factor are increased 3 years after Roux-en-Y gastric bypass (RYGB) for morbid obesity: implications for long-term neoplastic risk following RYGB. Gut.

[33] Tamboli RA, Hajri T, Jiang A, Marks-Shulman PA, Williams DB, Clements RH, Melvin W, Bowen BP, Shyr Y, Abumrad NN, Flynn CR (2011). Reduction in inflammatory gene expression in skeletal muscle from Roux-en-Y gastric bypass patients randomized to omentectomy. PLoS One.

[34] Bradley D, Conte C, Mittendorfer B, Eagon JC, Varela JE, Fabbrini E, Gastaldelli A, Chambers KT, Su X, Okunade A, Patterson BW, Klein S (2012). Gastric bypass and banding equally improve insulin sensitivity and β cell function. J Clin Invest.

[35] Barres R, Kirchner H, Rasmussen M, Yan J, Kantor FR, Krook A, Näslund E, Zierath JR (2013). Weight loss after gastric bypass surgery in human obesity remodels promoter methylation. Cell Rep.

[36] Kong LC, Tap J, Aron-Wisnewsky J, Pelloux V, Basdevant A, Bouillot JL, Zucker JD, Doré J, Clément K (2013). Gut microbiota after gastric bypass in human obesity: increased richness and associations of bacterial genera with adipose tissue genes. Am J Clin Nutr.

[37] Andersson DP, Eriksson Hogling D, Thorell A, Toft E, Qvisth V, Näslund E, Thörne A, Wirén M, Löfgren P, Hoffstedt J, Dahlman I, Mejhert N, Rydén M, Arner E, Arner P (2014). Changes in subcutaneous fat cell volume and insulin sensitivity after weight loss. Diabetes Care.

[38] Ferrer R, Pardina E, Rossell J, Baena-Fustegueras JA, Lecube A, Balibrea JM, Caubet E, González O, Vilallonga R, Fort JM, Peinado-Onsurbe J (2014). Decreased lipases and fatty acid and glycerol transporter could explain reduced fat in diabetic morbidly obese. Obesity (Silver Spring).

[39] Marambio A, Watkins G, Castro F, Riffo A, Zúñiga R, Jans J, Villanueva ME, Díaz G (2014). Changes in iron transporter divalent metal transporter 1 in proximal jejunum after gastric bypass. World J Gastroenterol.

[40] Albers PH, Bojsen-Møller KN, Dirksen C, Serup AK, Kristensen DE, Frystyk J, Clausen TR, Kiens B, Richter EA, Madsbad S, Wojtaszewski JF (2015). Enhanced insulin signaling in human skeletal muscle and adipose tissue following gastric bypass surgery. Am J Physiol Regul Integr Comp Physiol.

[41] Casselbrant A, Elias E, Fändriks L, Wallenius V (2015). Expression of tight-junction proteins in human proximal small intestinal mucosa before and after Roux-en-Y gastric bypass surgery. Surg Obes Relat Dis.

[42] Chen MZ, Hudson CA, Vincent EE, de Berker DA, May MT, Hers I, Dayan CM, Andrews RC, Tavaré JM (2015). Bariatric surgery in morbidly obese insulin resistant humans normalises insulin signalling but not insulin-stimulated glucose disposal. PLoS One.

[43] Coen PM, Menshikova EV, Distefano G, Zheng D, Tanner CJ, Standley RA, Helbling NL, Dubis GS, Ritov VB, Xie H, Desimone ME, Smith SR, Stefanovic-Racic M, Toledo FG, Houmard JA, Goodpaster BH (2015). Exercise and weight loss improve muscle mitochondrial respiration, lipid partitioning, and insulin sensitivity after gastric bypass surgery. Diabetes.

[44] Nascimento EB, Riedl I, Jiang LQ, Kulkarni SS, Näslund E, Krook A (2015). Enhanced glucose metabolism in cultured human skeletal muscle after Roux-en-Y gastric bypass surgery. Surg Obes Relat Dis.

[45] Nergård BJ, Lindqvist A, Gislason HG, Groop L, Ekelund M, Wierup N, Hedenbro JL (2015). Mucosal glucagon-like peptide-1 and glucose-dependent insulinotropic polypeptide cell numbers in the super-obese human foregut after gastric bypass. Surg Obes Relat Dis.

[46] Su X, Magkos F, Zhou D, Eagon JC, Fabbrini E, Okunade AL, Klein S (2015). Adipose tissue monomethyl branched-chain fatty acids and insulin sensitivity: effects of obesity and weight loss. Obesity (Silver Spring).

[47] Campbell LE, Langlais PR, Day SE, Coletta RL, Benjamin TR, De Filippis EA, Madura 2nd JA, Mandarino LJ, Roust LR, Coletta DK (2016). Identification of novel changes in human skeletal muscle proteome after Roux-en-Y gastric bypass surgery. Diabetes.

[48] González-Plaza JJ, Gutiérrez-Repiso C, García-Serrano S, Rodriguez-Pacheco F, Garrido-Sánchez L, Santiago-Fernández C, García-Arnés J, Moreno-Ruiz FJ, Rodríguez-Cañete A, García-Fuentes E (2016). Effect of Roux-en-Y gastric bypass-induced weight loss on the transcriptomic profiling of subcutaneous adipose tissue. Surg Obes Relat Dis.

[49] Severino A, Castagneto-Gissey L, Raffaelli M, Gastaldelli A, Capristo E, Iaconelli A, Guidone C, Callari C, Bellantone R, Mingrone G (2016). Early effect of Roux-en-Y gastric bypass on insulin sensitivity and signaling. Surg Obes Relat Dis.

[50] Hoffstedt J, Andersson DP, Eriksson Hogling D, Theorell J, Näslund E, Thorell A, Ehrlund A, Rydén M, Arner P (2017). Long-term protective changes in adipose tissue after gastric bypass. Diabetes Care.

[51] Sala P, Torrinhas RS, Fonseca DC, Heymsfield S, Giannella-Neto D, Waitzberg DL (2017). Type 2 diabetes remission after Roux-en-Y gastric bypass: evidence for increased expression of jejunal genes encoding regenerating pancreatic islet-derived proteins as a potential mechanism. Obes Surg.

[52] Hinkley JM, Zou K, Park S, Turner K, Zheng D, Houmard JA (2017). Roux-en-Y gastric bypass surgery enhances contraction-mediated glucose metabolism in primary human myotubes. Am J Physiol Endocrinol Metab.

[53] Hinkley JM, Zou K, Park S, Zheng D, Dohm GL, Houmard JA (2017). Differential acute and chronic responses in insulin action in cultured myotubes following from nondiabetic severely obese humans following gastric bypass surgery. Surg Obes Relat Dis.

[54] Day SE, Garcia LA, Coletta RL, Campbell LE, Benjamin TR, De Filippis EA, Madura 2nd JA, Mandarino LJ, Roust LR, Coletta DK (2017). Alterations of sorbin and SH3 domain containing 3 (SORBS3) in human skeletal muscle following Roux-en-Y gastric bypass surgery. Clin Epigenetics.

[55] Parker BM, Wu J, You J, Barnes DS, Yerian L, Kirwan JP, Schauer PR, Sessler DI (2017). Reversal of fibrosis in patients with nonalcoholic steatohepatosis after gastric bypass surgery. BMC Obes.

[56] Afshar S, Malcomson F, Kelly SB, Seymour K, Woodcock S, Mathers JC (2018). Biomarkers of colorectal cancer risk decrease 6 months after Roux-en-Y gastric bypass surgery. Obes Surg.

[57] Fonseca DC, Sala P, Singer J, Singer P, Torrinhas RS, Waitzberg DL (2018). Upregulation of ghrelin gene expression in the excluded stomach of obese women with type 2 diabetes after Roux-en-Y gastric bypass in the SURMetaGIT study. Obes Surg.

[58] Schwenger KJ, Fischer SE, Jackson T, Okrainec A, Allard JP (2018). In nonalcoholic fatty liver disease, Roux-en-Y gastric bypass improves liver histology while persistent disease is associated with lower improvements in waist circumference and glycemic control. Surg Obes Relat Dis.

[59] von Schönfels W, Beckmann JH, Ahrens M, Hendricks A, Röcken C, Szymczak S, Hampe J, Schafmayer C (2018). Histologic improvement of NAFLD in patients with obesity after bariatric surgery based on standardized NAS (NAFLD activity score). Surg Obes Relat Dis.

[60] Berisha SZ, Serre D, Schauer P, Kashyap SR, Smith JD (2011). Changes in whole blood gene expression in obese subjects with type 2 diabetes following bariatric surgery: a pilot study. PLoS One.

[61] Hulsmans M, Geeraert B, De Keyzer D, Mertens A, Lannoo M, Vanaudenaerde B, Hoylaerts M, Benhabilès N, Tsatsanis C, Mathieu C, Holvoet P (2012). Interleukin-1 receptor-associated kinase-3 is a key inhibitor of inflammation in obesity and metabolic syndrome. PLoS One.

[62] Ortega FJ, Mercader JM, Moreno-Navarrete JM, Nonell L, Puigdecanet E, Rodriquez-Hermosa JI, Rovira O, Xifra G, Guerra E, Moreno M, Mayas D, Moreno-Castellanos N, Fernández-Formoso JA, Ricart W, Tinahones FJ, Torrents D, Malagón MM, Fernández-Real JM (2015). Surgery-induced weight loss is associated with the downregulation of genes targeted by microRNAs in adipose tissue. J Clin Endocrinol Metab.

[63] Promintzer-Schifferl M, Prager G, Anderwald C, Mandl M, Esterbauer H, Shakeri-Leidenmühler S, Pacini G, Stadler M, Bischof MG, Ludvik B, Luger A, Krebs M (2011). Effects of gastric bypass surgery on insulin resistance and insulin secretion in nondiabetic obese patients. Obesity (Silver Spring).

[64] Salinari S, Bertuzzi A, Guidone C, Previti E, Rubino F, Mingrone G (2013). Insulin sensitivity and secretion changes after gastric bypass in normotolerant and diabetic obese subjects. Ann Surg.

[65] Bojsen-Møller KN, Dirksen C, Jørgensen NB, Jacobsen SH, Serup AK, Albers PH, Hansen DL, Worm D, Naver L, Kristiansen VB, Wojtaszewski JF, Kiens B, Holst JJ, Richter EA, Madsbad S (2014). Early enhancements of hepatic and later of peripheral insulin sensitivity combined with increased postprandial insulin secretion contribute to improved glycemic control after Roux-en-Y gastric bypass. Diabetes.

[66] Campos GM, Rabl C, Peeva S, Ciovica R, Rao M, Schwarz JM, Havel P, Schambelan M, Mulligan K (2010). Improvement in peripheral glucose uptake after gastric bypass surgery is observed only after substantial weight loss has occurred and correlates with the magnitude of weight lost. J Gastrointest Surg.

[67] Lima MM, Pareja JC, Alegre SM, Geloneze SR, Kahn SE, Astiarraga BD, Chaim EA, Geloneze B (2010). Acute effect of roux-en-y gastric bypass on whole-body insulin sensitivity: a study with the euglycemic-hyperinsulinemic clamp. J Clin Endocrinol Metab.

[68] Camastra S, Gastaldelli A, Mari A, Bonuccelli S, Scartabelli G, Frascerra S, Baldi S, Nannipieri M, Rebelos E, Anselmino M, Muscelli E, Ferrannini E (2011). Early and longer term effects of gastric bypass surgery on tissue-specific insulin sensitivity and beta cell function in morbidly obese patients with and without type 2 diabetes. Diabetologia.

[69] Dunn JP, Abumrad NN, Breitman I, Marks-Shulman PA, Flynn CR, Jabbour K, Feurer ID, Tamboli RA (2012). Hepatic and peripheral insulin sensitivity and diabetes remission at 1 month after Roux-en-Y gastric bypass surgery in patients randomized to omentectomy. Diabetes Care.

