# Supplementary material for: The Feasibility of Examining the Effects of Gastric Bypass Surgery on Intestinal Metabolism: Prospective, Longitudinal Mechanistic Clinical Trial
Source: JMIR Res Protoc. 2019 Jan 24;8(1):e12459. doi: 10.2196/12459 (PMC6483060; doi:10.2196/12459)
Supplement: Multimedia Appendix 1 [file resprot_v8i1e12459_app1.pdf]

Multimedia Appendix 1: Bariatric surgery studies involving analyses of tissue biopsies.

Study	Comparison groups	Tissue	Time point (postoperatively)	Key message
Ranlov et al, 1990 [23]	Before vs after RYGB	Liver	12 months	Occurrence of steatosis fell from 73% to 40% postoperatively, with concurrent improvements in inflammatory and granulomatous changes. No patients in the study had evidence of liver fibrosis.
Friedman et al, 1992 [24]	Before vs after RYGB	Skeletal muscle	15-24 months	Glucose disposal rate, as a measure of whole-body insulin sensitivity, increased in all patients after RYGB. Hepatic insulin sensitivity was not assessed. Ex-vivo study of muscle biopsies revealed increased insulin-induced 2-deoxyglucose transport after surgery.
De Almeida et al, 2006 [25]	Patients with NASH, before vs after RYGB	Liver	23.5±8.4 months	Steatosis, necroinflammatory activity, and hepatic fibrosis were improved after surgery. NAFLD resolved in 93.7% of patients.
Klein et al, 2006 [26]	Before vs after RYGB	Liver	1 year	RYGB reduced hepatic steatosis but did not change assessments of inflammation and fibrosis. However, expression of genes regulating hepatic

				fibrogenesis and inflammation were reduced.
Park et al, 2006 [22]	Before vs after RYGB	Skeletal muscle	1 year	After RYGB, muscle exhibited differential expression of genes linked to processes important for modulating insulin signaling and triglyceride synthesis.
Furuya et al, 2007 [27]	Patients with NAFLD, before vs after RYGB	Liver	2 years	NAFLD including steatohepatitis improved by 2 years after RYGB. Steatosis resolved in 84% and fibrosis in 75% of patients.
Gastaldi et al, 2007 [28]	Before vs after RYGB	Skeletal muscle	3 months and 1 year	Expression of PGC1α and its target mitofusin-2 (MFN2) in skeletal muscle were increased at both postoperative follow-up visits. Glucose disposal rate increased sequentially over the follow-up period, and MFN2 expression correlated positively with insulin sensitivity.
Sainsbury et al, 2008 [29]	Before vs after RYGB, vs lean controls	Rectum	6 months	Rectal tissue from obese patients exhibited increased mitosis, crypt area, and crypt branching as compared with lean controls, but mitosis was further increased after RYGB, with reduction in epithelial cell apoptosis.
Gregor et al,	Before vs after	Subcutaneous	1 year	Markers of ER stress in

2009 [30]	RYGB	adipose tissue, liver		adipose tissue and liver biopsies were reduced after RYGB. Clamp study of these patients showed that both glucose disposal and hepatic insulin sensitivity index improved after surgery, with a larger relative effect of the former.
Savu et al, 2009 [31]	Before vs after RYGB	Subcutaneous adipose tissue	2-5 months and during weight maintenance	After 20% postoperative weight loss, adipose expression of adiponectin and the adiponectin receptors, AdipoR1 and AdipoR2, were increased and remained increased with no further change at a later time point defined by steady-state weight loss achieved (defined as <2% change in body weight in a 3-month period).
Spak et al, 2010 [5]	Before vs after RYGB (some after conversion from VBG)	Jejunum	6-8 months	Protein expression of NADPH-oxidase, myeloperoxidase, and the Ang II type 1 receptor were increased in jejunum after surgery.
Kant et al, 2011 [32]	Before vs after RYGB	Rectum	3 years	Rectal epithelial cell mitosis and crypt size were increased 3 years after RYGB as compared with preoperative biopsies
Tamboli et	Before vs after	Skeletal muscle	6 and 12 months	Omentectomy in association

al, 2011 [33]	RYGB, ± omentectomy			with RYGB resulted in a greater reduction in expression of genes associated with muscle inflammation, as compared with RYGB alone. Genes driving protein turnover were altered in both groups postoperatively.
Bradley et al, 2012 [34]	Before vs after RYGB	Subcutaneous adipose tissue, skeletal muscle	After 20% weight loss achieved	Adipose tissue expression of proinflammatory macrophage cell surface markers was reduced after RYGB, and the expression of the anti-inflammatory cytokine, IL-10, was increased. Weight loss did not reduce intramyocellular DAG or ceramide content, suggesting that these metabolites do not mediate the improvements in peripheral insulin sensitivity observed in this study using hyperinsulinemic-euglycemic clamp.
Ahrens et al, 2013 [21]	Before vs after RYGB, vs lean controls	Liver	5-9 months	NAFLD was associated with a signature methylation pattern in liver tissue; this was partially reversed after RYGB.
Barres et al, 2013 [35]	Before vs after RYGB, vs lean controls	Skeletal muscle	6 months	Obesity was associated with a specific gene expression and methylation pattern in skeletal

				muscle, and this was normalized after RYGB.
Kong et al, 2013 [36]	Before vs after RYGB	Subcutaneous adipose tissue	3 months	This study correlated changes in the gut microbial profile after RYGB with changes in WAT gene expression, finding that variations in bacterial genera correlated with WAT gene expression changes.
Andersson et al, 2014 [37]	Before vs after RYGB	Subcutaneous adipose tissue	2 years	Reduction in abdominal subcutaneous fat cell volume correlates more strongly with improvements to insulin sensitivity than to fat mass.
Ferrer et al, 2014 [38]	Before vs after RYGB, vs lean controls	Subcutaneous adipose tissue	6 and 12 months	Activity of lipoprotein lipase and hormone sensitive lipase in subcutaneous adipose tissue were reduced after RYGB, approximating that of lean controls by 12 months postoperatively.
Marambio et al, 2014 [39]	Before vs after RYGB	Proximal jejunum	6 months	Immunohistochemistry showed increased staining for the iron transported DMT1 in enterocytes located at the tips of villi but less staining overall.
Albers et al, 2015 [40]	Before vs after RYGB	Subcutaneous adipose tissue, skeletal muscle	1 week, 3 months, 12 months	Insulin-induced phosphorylation of Akt was increased in both muscle and adipose 12 months after surgery, among other molecular changes interpreted

				to signify improved insulin sensitivity in both tissues.
Casselbrant et al, 2015 [41]	Before vs after RYGB	Jejunum	6-8 months	Jejunal mucosal surface area was increased after surgery. Increased protein-level expression of claudin-3 and claudin-4 and reduced expression of occludin and zona occludens-1 might signify reduced paracellular permeability.
Chen et al, 2015 [42]	Before vs after RYGB, vs lean controls	Skeletal muscle	6.8±1.2 months	Insulin-induced phosphorylation of skeletal muscle Akt at Thr308 and Ser473 was enhanced after RYGB and normalized relative to lean controls.
Coen et al, 2015 [43]	Before vs after RYGB; before vs after intensive exercise/health counseling program vs health education counseling only	Skeletal muscle	1-3 months, 7-9 months	In contrast to education alone, exercise increased mitochondrial respiration in muscle and reduced levels of certain sphingolipids. This was associated with improved whole-body insulin sensitivity as assessed by IV GTT.
Nascimento et al, 2015 [44]	Before vs after RYGB	Skeletal muscle	6 months	Myoblasts derived from muscle biopsies exhibited increased expression of myogenic markers. Study of cultured cells derived from

				these biopsies showed increased glycogen synthesis and increased insulin-stimulated phosphorylation of Akt and PKB, as markers of muscle insulin sensitivity.
Nergard et al, 2015 [45]	Before vs after RYGB	Proximal jejunum, stomach/pouch	12 months	Density of GLP-1, GIP, and PYY cells increased postoperatively. There was no change in villi length or in the density of other enteroendocrine cells including those expressing ghrelin, CCK, neurotensin, secretin, or serotonin. Neither mucosal height nor densities of ghrelin, histamine, serotonin, and somatostatin-producing cells were altered by RYGB.
Su et al, 2015 [46]	Before vs after RYGB, vs lean controls	Subcutaneous adipose tissue	1 year	Adipose tissue levels of monomethyl branched-chain amino acids were lower in obese vs lean patients but increased substantially after RYGB. This was associated with improved glucose disposal during the clamp.
Campbell et al, 2016 [47]	Before vs after RYGB, vs lean controls	Skeletal muscle	3 months	Obesity affected proteomic and gene expression patterns in muscle; this was partially reversed by RYGB.
González-	Before vs after	Subcutaneous	2 years	Weight loss was stable by 2

Plaza et al, 2016 [48]	RYGB	adipose tissue		years postoperatively. Adipose gene expression revealed an induction, relative to the preoperative time point, in genes involved in regulation of lipid metabolism and downregulation of immune/inflammatory processes.
Severino et al, 2016 [49]	Before vs after RYGB	Skeletal muscle	4 weeks	Insulin sensitivity improved after RYGB as compared with preoperatively but was not normalized as compared with healthy control patients. Study of muscle biopsies demonstrated hyperphosphorylation of pAKT on Ser473 residues preoperatively as compared with controls; this normalized after surgery and is hypothesized to underlie the improved insulin resistance observed in this study.
Hoffstedt et al, 2017 [50]	Before vs after RYGB	Subcutaneous adipose tissue	2 and 5 years	Adipose cell size and lipolysis decreased by the 2-year postoperative time point, with no change in adipocyte number. By 5 years after surgery, average BMI was higher and adipocytes were more abundant but unchanged in size. Adipose-secreted adiponectin levels were

				increased 2 years after surgery and did not change between the 2- and 5-year postoperative time points.
Sala et al, 2017 [51]	Before vs after RYGB	Duodenum, jejunum, and ileum	3 months	Expression of regenerating pancreatic islet-derived protein-encoding genes was increased in the intestine after RYGB. This phenomenon is postulated to contribute to T2D remission after surgery
Hinkley et al, 2017 [52]	Before vs after RYGB	Skeletal muscle	1 month	After RYGB, contraction enhanced insulin-stimulated glycogen synthesis and basal glucose oxidation in skeletal muscle
Hinkley et al, 2017 [53]	Before vs after RYGB	Skeletal muscle	1 and 7 months	Differentiated myotubes developed from muscle biopsies demonstrated increased insulin-stimulated glycogen synthesis and glucose oxidation after surgery. In addition, muscle glycogen levels were lower and phosphorylation of acetyl coA carboxylase 2 was increased in muscle 1 month after surgery, whereas both returned to baseline levels by 7 months postoperatively. PGC1α protein content was increased in myotubes 7 months after RYGB.

Day et al, 2017 [54]	Before vs after RYGB	Skeletal muscle	3 months	Reduced methylation of the *SORBS3* gene promotor was demonstrated, in tandem with increased expression of the transcript and altered reporter gene expression in vitro. Changes in *SORBS3* expression correlated with clinical outcomes including obesity measures and fasting insulin levels in patients.
Parker et al, 2017 [55]	Before vs after RYGB	Liver	Not specified; for larger study, average of 487±86 days	Nine of 13 patients with fatty liver disease before RYGB exhibited normalization after weight loss. The other 4 patients exhibited stabilization or improvement in histology.
Afshar et al, 2018 [56]	Before vs after RYGB	Rectal mucosa	Median of 6.5 months	RYGB did not increase expression of proinflammatory genes in rectal mucosa, and COX-1 expression was reduced. Additionally, the number of mitoses per crypt was reduced in rectal biopsies after surgery.
Fonseca et al, 2018 [57]	Before vs after RYGB	Stomach	3 months	Ghrelin gene expression was increased in stomach tissue after RYGB, without increasing plasma ghrelin levels.
Schwenger	Before vs after	Liver	12 months	RYGB improved histologic

et al, 2018 [58]	RYGB			evidence of NAFLD.
Von Schönfels et al, 2018 [59]	Before vs after RYGB	Liver	Median of 192 days	Retrospective analysis of liver biopsy samples collected in patients before and after RYGB demonstrated improvement in NAFLD score based on liver histology.

References:

23. Ranlov I, Hardt F. Regression of liver steatosis following gastroplasty or gastric bypass for morbid obesity. *Digestion.* 1990;47(4):208-214.

24. Friedman JE, Dohm GL, Leggett-Frazier N, et al. Restoration of insulin responsiveness in skeletal muscle of morbidly obese patients after weight loss. Effect on muscle glucose transport and glucose transporter GLUT4. *The Journal of clinical investigation.* Feb 1992;89(2):701-705.

25. de Almeida SR, Rocha PR, Sanches MD, et al. Roux-en-Y gastric bypass improves the nonalcoholic steatohepatitis (NASH) of morbid obesity. *Obesity surgery.* Mar 2006;16(3):270-278.

26. Klein S, Mittendorfer B, Eagon JC, et al. Gastric bypass surgery improves metabolic and hepatic abnormalities associated with nonalcoholic fatty liver disease. *Gastroenterology.* May 2006;130(6):1564-1572.

22. Park JJ, Berggren JR, Hulver MW, Houmard JA, Hoffman EP. GRB14, GPD1, and GDF8 as potential network collaborators in weight loss-induced improvements in insulin action in human skeletal muscle. *Physiological genomics.* Oct 11 2006;27(2):114-121.

27. Furuya CK, Jr., de Oliveira CP, de Mello ES, et al. Effects of bariatric surgery on nonalcoholic fatty liver disease: preliminary findings after 2 years. *Journal of gastroenterology and hepatology.* Apr 2007;22(4):510-514.

28. Gastaldi G, Russell A, Golay A, et al. Upregulation of peroxisome proliferator-activated receptor gamma coactivator gene (PGC1A) during weight loss is related to insulin sensitivity but not to energy expenditure. *Diabetologia.* Nov 2007;50(11):2348-2355.

29. Sainsbury A, Goodlad RA, Perry SL, Pollard SG, Robins GG, Hull MA. Increased colorectal epithelial cell proliferation and crypt fission associated with obesity and roux-en-Y gastric bypass. *Cancer epidemiology, biomarkers & prevention : a publication of the American Association for Cancer Research, cosponsored by the American Society of Preventive Oncology.* Jun 2008;17(6):1401-1410.

30. Gregor MF, Yang L, Fabbrini E, et al. Endoplasmic reticulum stress is reduced in tissues of obese subjects after weight loss. *Diabetes.* Mar 2009;58(3):693-700.

31. Savu MK, Phillips SA, Oh DK, et al. Response of adiponectin and its receptors to changes in metabolic state after gastric bypass surgery: dissociation between adipose tissue expression and circulating levels. *Surgery for obesity and related diseases : official journal of the American Society for Bariatric Surgery.* Mar-Apr 2009;5(2):172-180.

5. Spak E, Bjorklund P, Helander HF, et al. Changes in the mucosa of the Roux-limb after gastric bypass surgery. *Histopathology.* Nov 2010;57(5):680-688.

32. Kant P, Sainsbury A, Reed KR, et al. Rectal epithelial cell mitosis and expression of macrophage migration inhibitory factor are increased 3 years after Roux-en-Y gastric bypass (RYGB) for morbid obesity: implications for long-term neoplastic risk following RYGB. *Gut*. Jul 2011;60(7):893-901.

33. Tamboli RA, Hajri T, Jiang A, et al. Reduction in inflammatory gene expression in skeletal muscle from Roux-en-Y gastric bypass patients randomized to omentectomy. *PloS one*. 2011;6(12):e28577.

34. Bradley D, Conte C, Mittendorfer B, et al. Gastric bypass and banding equally improve insulin sensitivity and beta cell function. *The Journal of clinical investigation*. Dec 2012;122(12):4667-4674.

21. Ahrens M, Ammerpohl O, von Schonfels W, et al. DNA methylation analysis in nonalcoholic fatty liver disease suggests distinct disease-specific and remodeling signatures after bariatric surgery. *Cell metabolism*. Aug 06 2013;18(2):296-302.

35. Barres R, Kirchner H, Rasmussen M, et al. Weight loss after gastric bypass surgery in human obesity remodels promoter methylation. *Cell reports*. Apr 25 2013;3(4):1020-1027.

36. Kong LC, Tap J, Aron-Wisnewsky J, et al. Gut microbiota after gastric bypass in human obesity: increased richness and associations of bacterial genera with adipose tissue genes. *The American journal of clinical nutrition*. Jul 2013;98(1):16-24.

37. Andersson DP, Eriksson Hogling D, Thorell A, et al. Changes in subcutaneous fat cell volume and insulin sensitivity after weight loss. *Diabetes care*. Jul 2014;37(7):1831-1836.

38. Ferrer R, Pardina E, Rossell J, et al. Decreased lipases and fatty acid and glycerol transporter could explain reduced fat in diabetic morbidly obese. *Obesity*. Nov 2014;22(11):2379-2387.

39. Marambio A, Watkins G, Castro F, et al. Changes in iron transporter divalent metal transporter 1 in proximal jejunum after gastric bypass. *World journal of gastroenterology*. Jun 07 2014;20(21):6534-6540.

40. Albers PH, Bojsen-Moller KN, Dirksen C, et al. Enhanced insulin signaling in human skeletal muscle and adipose tissue following gastric bypass surgery. *American journal of physiology. Regulatory, integrative and comparative physiology*. Sep 2015;309(5):R510-524.

41. Casselbrant A, Elias E, Fandriks L, Wallenius V. Expression of tight-junction proteins in human proximal small intestinal mucosa before and after Roux-en-Y gastric bypass surgery. *Surgery for obesity and related diseases : official journal of the American Society for Bariatric Surgery*. Jan-Feb 2015;11(1):45-53.

42. Chen MZ, Hudson CA, Vincent EE, et al. Bariatric surgery in morbidly obese insulin resistant humans normalises insulin signalling but not insulin-stimulated glucose disposal. *PloS one*. 2015;10(4):e0120084.

43. Coen PM, Menshikova EV, Distefano G, et al. Exercise and Weight Loss Improve Muscle Mitochondrial Respiration, Lipid Partitioning, and Insulin Sensitivity After Gastric Bypass Surgery. *Diabetes*. Nov 2015;64(11):3737-3750.

44. Nascimento EB, Riedl I, Jiang LQ, Kulkarni SS, Naslund E, Krook A. Enhanced glucose metabolism in cultured human skeletal muscle after Roux-en-Y gastric bypass surgery. *Surgery for obesity and related diseases : official journal of the American Society for Bariatric Surgery*. May-Jun 2015;11(3):592-601.

45. Nergard BJ, Lindqvist A, Gislason HG, et al. Mucosal glucagon-like peptide-1 and glucose-dependent insulinotropic polypeptide cell numbers in the super-obese human foregut after gastric bypass. *Surgery for obesity and related diseases : official journal of the American Society for Bariatric Surgery*. Nov-Dec 2015;11(6):1237-1246.

46. Su X, Magkos F, Zhou D, et al. Adipose tissue monomethyl branched-chain fatty acids and insulin sensitivity: Effects of obesity and weight loss. *Obesity*. Feb 2015;23(2):329-334.

47. Campbell LE, Langlais PR, Day SE, et al. Identification of Novel Changes in Human Skeletal Muscle Proteome After Roux-en-Y Gastric Bypass Surgery. *Diabetes.* Sep 2016;65(9):2724-2731.

48. Gonzalez-Plaza JJ, Gutierrez-Repiso C, Garcia-Serrano S, et al. Effect of Roux-en-Y gastric bypass-induced weight loss on the transcriptomic profiling of subcutaneous adipose tissue. *Surgery for obesity and related diseases : official journal of the American Society for Bariatric Surgery.* Feb 2016;12(2):257-263.

49. Severino A, Castagneto-Gissey L, Raffaelli M, et al. Early effect of Roux-en-Y gastric bypass on insulin sensitivity and signaling. *Surgery for obesity and related diseases : official journal of the American Society for Bariatric Surgery.* Jan 2016;12(1):42-47.

50. Hoffstedt J, Andersson DP, Eriksson Hogling D, et al. Long-term Protective Changes in Adipose Tissue After Gastric Bypass. *Diabetes care.* Jan 2017;40(1):77-84.

51. Sala P, Torrinhas RS, Fonseca DC, Heymsfield S, Giannella-Neto D, Waitzberg DL. Type 2 Diabetes Remission After Roux-en-Y Gastric Bypass: Evidence for Increased Expression of Jejunal Genes Encoding Regenerating Pancreatic Islet-Derived Proteins as a Potential Mechanism. *Obesity surgery.* Apr 2017;27(4):1123-1127.

52. Hinkley JM, Zou K, Park S, Turner K, Zheng D, Houmard JA. Roux-en-Y gastric bypass surgery enhances contraction-mediated glucose metabolism in primary human myotubes. *American journal of physiology. Endocrinology and metabolism.* Aug 1 2017;313(2):E195-E202.

53. Hinkley JM, Zou K, Park S, Zheng D, Dohm GL, Houmard JA. Differential acute and chronic responses in insulin action in cultured myotubes following from nondiabetic severely obese humans following gastric bypass surgery. *Surgery for Obesity and Related Diseases*. 2017;13(11):1853-1862.

54. Day SE, Garcia LA, Coletta RL, et al. Alterations of sorbin and SH3 domain containing 3 (SORBS3) in human skeletal muscle following Roux-en-Y gastric bypass surgery. *Clinical epigenetics.* 2017;9:96.

55. Parker BM, Wu J, You J, et al. Reversal of fibrosis in patients with nonalcoholic steatohepatosis after gastric bypass surgery. *BMC obesity.* 2017;4:32.

56. Afshar S, Malcomson F, Kelly SB, Seymour K, Woodcock S, Mathers JC. Biomarkers of Colorectal Cancer Risk Decrease 6 months After Roux-en-Y Gastric Bypass Surgery. *Obesity surgery.* Apr 2018;28(4):945-954.

57. Fonseca DC, Sala P, Singer J, Singer P, Torrinhas RS, Waitzberg DL. Upregulation of Ghrelin Gene Expression in the Excluded Stomach of Obese Women with Type 2 Diabetes After Roux-en-Y Gastric Bypass in the SURMetaGIT Study. *Obesity surgery.* Mar 2018;28(3):877-880.

59. Schwenger KJP, Fischer SE, Jackson T, Okrainec A, Allard JP. In nonalcoholic fatty liver disease, Roux-en-Y gastric bypass improves liver histology while persistent disease is associated with lower improvements in waist circumference and glycemic control. *Surgery for obesity and related diseases : official journal of the American Society for Bariatric Surgery.* Sep 2018;14(9):1233-1239.

59. von Schonfels W, Beckmann JH, Ahrens M, et al. Histologic improvement of NAFLD in patients with obesity after bariatric surgery based on standardized NAS (NAFLD activity score). *Surgery for obesity and related diseases : official journal of the American Society for Bariatric Surgery.* Oct 2018;14(10):1607-1616.
